# Immediate Versus Delayed Hip Arthroscopy for Femoroacetabular Impingement: An Expected Value Decision Analysis

**DOI:** 10.5435/JAAOSGlobal-D-20-00206

**Published:** 2020-12-08

**Authors:** Manish P. Mehta, Michael A. Hoffer-Hawlik, Michaela O'Connor, T. Sean Lynch

**Affiliations:** From the Department of Orthopedic Surgery, Columbia University Irving Medical Center, New York, NY.

## Abstract

**Introduction:**

Hip arthroscopy is an increasingly used surgical procedure for both intra- and extra-articular hip pathologies, including femoroacetabular impingement (FAI). Although the arthroscopic approach is known to be preferable to open, the optimal timing of such intervention is unclear. The purpose of this study was to carry out an expected value decision analysis of immediate versus delayed hip arthroscopy for FAI. Its hypothesis is immediate hip arthroscopy is the preferable treatment option.

**Methods:**

An expected value decision analysis was implemented to systematize the decision-making process between immediate and delayed hip arthroscopies. A decision tree was created with options for immediate and delayed surgeries with utilities characterizing each state obtained from surveying 70 patients. Fold-back analysis was then carried out, calculating expected values by multiplying the utility of each health outcome by the probability of that outcome. Corresponding expected values were then summed to “fold back” the decision tree one layer at a time. This was repeated until overall expected values (0 to 100) for immediate and delayed hip arthroscopies resulted with the higher value indicating the preferable option.

**Results:**

Fold-back analysis demonstrated that immediate hip arthroscopy is the preferred treatment for FAI over delayed with expected values of 78.27 and 72.63, respectively. Restoration of good function after hip arthroscopy was the most notable contributor to this difference. Immediate hip arthroscopy remained superior even as vast adjustments to preoperative physical function were made in one-way sensitivity analysis. Complications of hip arthroscopy leading to total hip arthroplasty were the least notable contributors to overall expected values.

**Discussion:**

This study confirms that immediate surgery is the preferred option when using decision-making analysis combining patient-reported utilities of health outcomes and the probabilities of those outcomes from the literature. This is consistent across a range of estimates of poor function in both the delayed and immediate surgery arms.

Femoroacetabular impingement (FAI) is an increasingly recognized cause of hip pain and dysfunction necessitating definitive treatment.^1^ In addition to pain and diminished function, repeated microtrauma instigated by premature contact between the proximal femur and acetabulum is theorized to contribute to the eventual development of hip osteoarthritis, likely by way of wear of the labrum and articular cartilage.^2^ Factors affecting the likelihood and rate of this progression are not yet well understood.^3,4^

Hip arthroscopy is currently considered the standard treatment for FAI and for the correction of both labral and articular cartilage pathologies.^5^ The use of hip arthroscopy has increased almost fivefold over a period of eight years from 2005 to 2013 and has supplanted more invasive treatment methods.^6^ The arthroscopic approach leads to reduced surgical site morbidity and faster recovery times compared with open procedures such as surgical hip dislocation.^7-9^

In addition to correcting anatomic abnormalities, hip arthroscopy has been shown to be a safe and successful intervention in restoring function to patients with low complication rates. Minkara et al^10^ found in a review of 1981 cases that all patient-reported outcomes (PROs) improved after surgical intervention and also determined that the procedure has a low rate of a reoperation (5.5%) and complications (1.7%). O'Connor et al^11^ found that the average rate of return to play was 84.6% and that professional athletes may return to play at rates as high as 93.3%. In addition, a comparison of arthroscopic treatment with nonoperative management for FAI has demonstrated clinically meaningful improvements in patient-reported hip function and quality of life in the arthroscopic population.^12^

Although hip arthroscopy has become the standard of care for the treatment of FAI, the optimal timing of the intervention remains controversial. This has been compounded by the fact that, in many cases, insurance guidelines require patients to demonstrate months of symptoms and undergo a trial of nonsurgical management before surgery. It has been shown that patients undergoing hip arthroscopy within months of symptom onset demonstrate improved hip functionality and reduced likelihood of revision surgery,^13^ but further study of patient-reported preferences to determine the optimal timing of the intervention is necessary. Expected value decision analysis is a rigorous method for combining patient preferences with the likelihoods of health outcomes that has previously been used to guide medical care and decision-making, including within orthopaedic surgery.^14,15,16,17^ The purpose of this study was to carry out an expected value decision analysis of immediate versus delayed hip arthroscopy for FAI with the hypothesis that immediate hip arthroscopy is the preferable treatment option.

## Methods

### Overview

Expected value decision analysis is a quantitative approach to determining an optimal treatment decision in the event of limited clinical evidence.^15^ This study did a standard 5-step decision analysis that comprised (1) constructing a clinical decision model of health states, (2) assigning outcome probabilities to each of these states, (3) determining patient-derived utility values for each state, (4) conducting a fold-back analysis to sum the products of probabilities and utilities to determine the expected values for each treatment option, and (5) doing a one-way sensitivity analysis on the probability and value estimates.

### Step 1: Model

**Figure 1:**
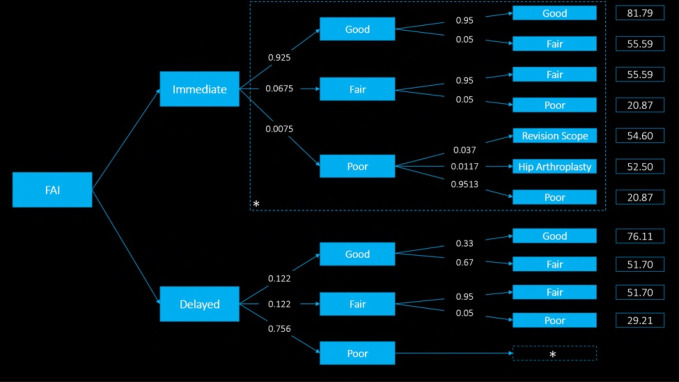
Decision tree demonstrating immediate versus delayed arthroscopic surgery. Values over arrows represent probabilities of the indicated outcome. Boxed values represent average patient-determined utilities for each state. “Good,” “fair,” and “poor” denote the level of function experienced by the patient after the specified treatment course. The asterisk (*) in the delayed treatment arm—after “poor” function—represents the fact that all patients with poor function underwent arthroscopic treatment with the same outcomes and probabilities as in the immediate treatment arm.

An immediate versus delayed treatment for FAI decision tree was formulated (Figure 1). In both the immediate and delayed cases, hip arthroscopy and nonsurgical treatment, respectively, were given the possibility of leading to good, fair, and poor outcomes. A *good outcome* was defined as the ability to participate in activities of daily living and sporting activities with minimal to no pain. A *fair outcome* was defined as the ability to participate in activities of daily living but not sport because of pain or limitations in motion. A *poor outcome* was defined as the inability to participate in activities of daily living and sport because of pain or limitations in motion.

In both the immediate and delayed treatment arms, good function could remain good over time or decline to fair function, whereas fair function could similarly remain fair over time or decline to poor function. In the immediate treatment arm, poor outcomes could either lead to a revision scope, revision to hip arthroplasty, or patients' opting for no further treatment and remaining with poor function. In the delayed treatment arm, all patients with poor function underwent arthroscopic treatment with the same outcomes and probabilities as in the immediate treatment arm, represented by the asterisk (*) in the model. Probabilities were assigned to transitions between health states based on literature, and utilities were assigned to health states based on patient input, each as described below. Based on the literature from which probabilities were obtained, the delayed treatment arm represented a difference in time to surgery of 10 weeks.^18^

### Step 2: Probabilities

A systematic review of literature was done using PubMed and MEDLINE with key words “hip arthroscopy,” “femoroacetabular impingement,” and “outcome” and “nonoperative,” “femoroacetabular impingement,” and “outcome.” Case reports, non-English publications, and clinical series with less than six months of follow-up were excluded. Where available, probabilities were directly correlated to state transitions based on data found in systematic literature reviews, randomized controlled trials, or population-level data analysis. In some cases where data were otherwise unavailable, previously published theoretical models of hip arthroscopy outcomes were used (Table 1). To apply transition probabilities for fair and poor function states after arthroscopy, rates of minor and major complications were used with minor corresponding to a fair health state and major corresponding to poor (Table 2; Figure 1, values over arrows).^18,19,20,21^

**Table 1:** Probabilities and Associated References for Health States Represented in the Decision Tree Model

Parameter	Probability	Reference	Study Design
Immediate			
Transition to good health state	0.925	Mather et al^19^	Economic and decision analysis
Transition to fair health state	0.0675	Mather et al^19^	Economic and decision analysis
Transition to poor health state	0.0075	Mather et al^19^	Economic and decision analysis
Conversion from poor health state to revision scope	0.037	Kester et al^20^	Case-control study
Conversion from poor health state to THA	0.117	Schairer et al^21^	Retrospective comparative study
Delayed			
Transition to good health state	0.112	Mansell et al^18^	Randomized controlled trial
Transition to fair health state	0.112	Mansell et al^18^	Randomized controlled trial
Transition to poor health state	0.756	Mansell et al^18^	Randomized controlled trial
Symptom recurrence after initial successful nonsurgical treatment	0.67	Mather et al^19^	Economic and decision analysis
All			
Progression of symptom severity (annual)	0.05	Mather et al^19^	Economic and decision analysis

THA = total hip arthroplasty

**Table 2:** Probabilities, Utilities, and Expected Values for Each Health Outcome in Cases of Both Immediate and Delayed Hip Arthroscopy

Health Outcome	Probability	Utility	Expected Value
Immediate			
Good to good function	0.95	81.79	77.70
Good to fair function	0.05	55.59	2.78
Fair to fair function	0.95	55.59	52.81
Fair to poor function	0.05	20.87	1.04
Poor function to revision scope	0.0370	54.60	2.02
Poor function to THA	0.0117	52.50	6.14
Poor to poor function	0.9513	20.87	17.66
Delayed			
Good to good function	0.33	76.11	25.12
Good to fair function	0.67	51.70	34.64
Fair to fair function	0.95	51.70	49.12
Fair to poor function	0.05	29.21	1.46
Poor function to arthroscopic intervention			59.17

THA = total hip arthroplasty

### Step 3: Utilities

After institutional review board approval, 70 patients from the practice of a single surgeon at a tertiary referral center in a high-volume urban center were sequentially surveyed to determine their health-state preferences and to calculate utilities of each health state, similar to a previously published study.^14^ To adequately represent the diversity of perspectives of patients with FAI based on differential activity levels and experience of symptomology, patients were only required to have had a history of FAI. In accordance with accepted methods, patients were asked to hypothetically rate on a scale of—“0 (death or worst health state imaginable) to 100 (perfect health)”—a variety of health states corresponding to those in the above-described model.^22^ Definitions of good, fair, and poor functions were also given as noted above. These values were then averaged to represent the utility of each terminal health state in the model (Table 2; Figure 1, boxed values).

### Step 4: Fold-back Analysis

With the decision tree complete, fold-back analysis was then carried out to determine whether immediate or delayed treatment is superior in the management of FAI. For each terminal health state, the literature-derived probability of transitioning to that state was multiplied by the patient-determined utility of being in that state. These values were then summed to calculate the combined expected utility of the original state that led into each of those terminal states. For example, the expected utility of good function after immediate treatment was calculated as the probabilities of remaining with good function and progressing to fair function each multiplied by the corresponding utilities of being in those states and then summed. This process was then repeated to calculate expected values for immediate treatment and delayed treatment with the larger value denoting the preferable option. Fold-back analysis was carried out in Microsoft Excel 2016 (Microsoft).

### Step 5: Sensitivity Analysis

To examine the significance of rates of poor function on the expected values for immediate and delayed treatment, one-way sensitivity analysis was also done by adjusting rates of poor function in the delayed and then the immediate surgery arms, repeating the analysis for each, and comparing overall expected values. Sensitivity analysis was carried out in Microsoft Excel 2016 (Microsoft).

## Results

### Probabilities

Probabilities of transitioning into each health state are listed in Table 2 and above the arrows in Figure 1. The probabilities of resulting in each terminal health state after immediate surgery are as follows: 87.88% good function, 11.04% fair function, 0.97% poor function, 0.028% revision arthroscopy, and 0.088% total hip arthroplasty (THA). Those after delayed surgery are as follows: 4.03% good function, 19.76% fair function, and 76.21% poor function.

### Utilities

Utilities for each of the terminal health states are listed in Table 2 and shown as boxed values in Figure 1. Health states with the greatest utilities included arthroscopic treatment for FAI leading to good function and nonsurgical treatment for FAI leading to good hip function with mean utilities of 81.79 and 76.11, respectively. Health states with the lowest utilities included arthroscopic treatment for FAI leading to poor hip function and requiring a second surgery and nonsurgical treatment for FAI leading to poor hip function and eventually requiring surgery with utilities of 20.87 and 29.21, respectively.

### Fold-back Analysis

Fold-back analysis demonstrated that immediate hip arthroscopy is the preferred treatment for FAI over delayed with overall expected values of 78.27 and 72.63, respectively. Restoration of good function after hip arthroscopy was the most notable contributor to this difference with an expected value of 77.70 for immediate surgery and only 25.12 for delayed. Complications of hip arthroscopy leading to THA were the least notable contributor with an expected value of just 6.14 (Table 2).

### Sensitivity Analysis

**Figure 2:**
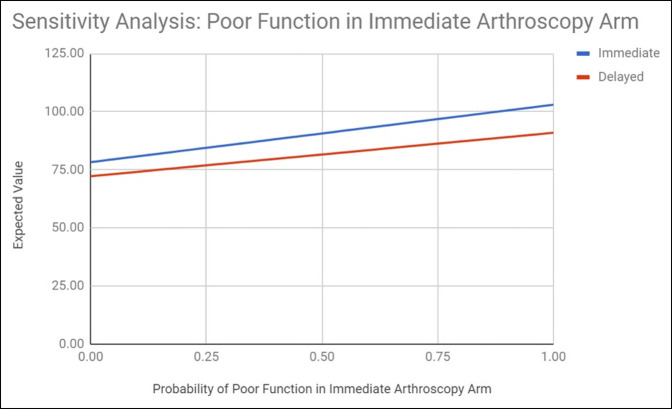
Sensitivity analysis demonstrating the effect of variability of the value of probability of poor function in the immediate arthroscopy arm on the resultant immediate and delayed treatment expected values.

**Figure 3:**
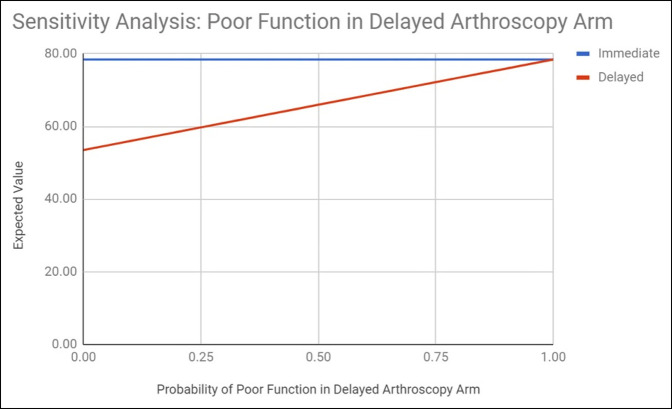
Sensitivity analysis demonstrating the effect of variability of the value of probability of poor function in the delayed arthroscopy arm on the resultant immediate and delayed treatment expected values.

Immediate hip arthroscopy remained the superior option despite vast adjustments to rates of poor function in the delayed and immediate surgery arms in one-way sensitivity analysis. When the rate of poor function in the immediate surgery arm was varied, the expected value of both delayed and immediate intervention increased linearly from 72.19 and 78.23 (poor function rate 0) to 90.90 and 102.97 (poor function rate 1.00), respectively (Figure 2). When the rate of poor function in the delayed surgery arm was varied, the expected value of delayed intervention increased linearly from 53.49 (poor function rate 0) to 78.41 (poor function rate 1.00), whereas the expected value of immediate intervention remained unaffected at 78.41 (Figure 3). In all cases, however, immediate intervention retained a larger expected value and so remained the preferable option.

## Discussion

This expected value decision analysis illustrates that immediate hip arthroscopy is the preferred management option over delayed treatment, which is consistent even as the probability of patients with preoperative poor function varied in both the immediate and delayed treatment arms. These findings agree with the literature, especially with regard to the effects of symptom duration on surgical outcome. Byrd and Jones^23^ found that increased symptom duration before hip arthroscopy was indicative of worse PRO scores after surgical intervention. In a study of patients in a national health care system, patients who were waitlisted for surgery had notably lower PRO scores than patients who were able to schedule surgery quickly.^12^ In a system with private payers, Dierckman et al^24^ likewise determined that increased symptom times were correlated with lower PRO scores on a logarithmic scale. Aprato et al found that patients who had delayed arthroscopy with preoperative symptoms lasting greater than three years had notably worse outcomes than those with shorter symptom durations. Moreover, within the short symptom cohort, those patients who underwent surgery less than six months after symptom onset had notably better postoperative outcomes than those waiting longer.^13^ Kunze et al^25^ similarly found that surgical intervention within three to six months of symptom onset was associated with superior postoperative outcomes. In fact, combining literature-based outcome data with directly obtained patient preferences, this study demonstrates that immediate arthroscopic treatment is preferable over delayed treatment even 10 weeks later.

Supporting this recommendation is that immediate surgical intervention is itself not quite immediate. Patients with conditions appropriate for treatment with hip arthroscopy often see numerous medical providers before consultation with a hip arthroscopist. Kahlenberg et al^26^ found that 82.1% of patients saw more than one healthcare provider, averaging four visits before surgical evaluation and costing an average of $315.05 per patient. Diagnosis of a labral tear did not occur until 32 months (2.67 years) after symptom onset. During this time, most patients had already begun conservative treatment before seeing a surgeon with an average of 3.1 treatments tried by each patient, including activity restriction, anti-inflammatory medications, and physical therapy. The time lost to nonsurgical evaluation and treatment directly affects postoperative outcomes. Basques et al^27^ reported that patients undergoing arthroscopic treatment with greater than two years of symptoms had notably worse PRO scores and higher revision surgery rates than those with shorter symptom durations. Patients surveyed in this study noted arthroscopic treatment leading to poor hip function and requiring a second surgery as their least preferred outcome (ie, the health state with the lowest utility). Thus, additional delay after surgical evaluation is deleterious.

Arguing against immediate intervention is the apparent cost of surgical intervention compared with nonsurgical management. Indeed, treating FAI is expensive with associated costs averaging $2,456.97.^26^ However, hip arthroscopy is actually a cost-effective—and sometimes even cost-saving—procedure. Lodhia et al^28^ found that although hip arthroscopy seems more expensive initially, it is actually cost-effective for 94.5% of patients. Mather et al^19^ also determined that society saves an average $67,418 per hip arthroscopy patient over the course of 10 years and that hip arthroscopy is the more cost-effective option 99% of the time. Furthermore, arthroscopic surgery of the hip is cost-effective when compared with salvage THA for patients with end-stage degenerative disease and even becomes cost-saving if it staves off THA conversion for at least 16 years.^29,30^ Along these lines, the procedure is considered cost-effective in all instances by the UK's National Institute for Health and Care Excellence.^31^

Within this context, this study's findings suggest that immediate treatment with hip arthroscopy holds broad policy implications regarding prioritizing patient preferences. Regulatory and insurance barriers that limit access to orthopaedic treatment may prevent patients from undergoing hip arthroscopy per their desired timing and lead to feared results. Oft-delayed presentations long after onset of symptoms and the extra inconvenience and cost of nonorthopaedic evaluations further impact patient satisfaction and post-operative outcomes. This study strengthens previous research supporting that early referral to orthopaedic care and timely surgical treatment are important factors for both payers and individual patients to ensure patient-centered care.^32^

Limitations of this study include the fact that although the model's utilities were directly acquired from patients treated at our institution, its outcome probabilities were derived from studies across multiple centers, an intrinsic limitation of this type of analysis and represented in the published literature.^14^ In addition, capturing utilities from a single practice introduces sampling bias and may affect the external validity of our model. However, our practice being based within a tertiary referral center in a large metropolitan area with a diverse patient population and all patients with a diagnosis of FAI being sequentially included in the study with no further restrictions on the surveyed population provide some mitigation. Finally, this study's model assumes a limited number of discrete potential health states for simplicity, whereas clinical outcomes are rarely able to be so clearly delineated. The intrinsic limitation of simplifying assumptions is a requisite part of any such analysis. Nonetheless, as previously noted, expected value decision analysis remains a useful tool in guiding clinical decision-making.^14,15,16,17^

This study's findings further the literature in support of immediate hip arthroscopy over delayed treatment for FAI. Even when rates of poor function in the delayed and immediate surgery arms were adjusted in one-way sensitivity analysis, immediate treatment remained the superior option. These robust results combine patient-reported utilities and literature-based likelihoods of potential outcomes to build on the current literature-supported consensus for arthroscopic management over conservative care by arguing for immediate arthroscopic intervention rather than delayed for FAI.
